# Impact of Prematurity on Metabolic Maturation

**DOI:** 10.1021/acs.jproteome.5c00640

**Published:** 2026-03-23

**Authors:** Kate Pearse, Aneurin Young, Mark J. Johnson, R. Mark Beattie, Jonathan R. Swann, Luise V. Marino

**Affiliations:** † School of Human Development and Health, Faculty of Medicine, 7423 University of Southampton, Southampton SO16 6YD, UK; ‡ Department of Neonatal Medicine, Princess Anne Hospital, University Hospital Southampton NHS Foundation Trust, Southampton SO16 5YA, UK; § Paediatric Gastroenterology, Southampton Children’s Hospital, 7425 University Hospital Southampton NHS Foundation Trust, Southampton SO16 6YD, UK; ∥ Southampton NIHR Biomedical Research Centre, University Hospital Southampton NHS Foundation Trust, Southampton SO16 6YD, UK; ⊥ Faculty of Health Science, University of Southampton, Southampton SO17 1BJ, UK; # Department of Metabolism, Digestion and Reproduction, Imperial College London, London SW7 2AZ, UK; ∇ Research & Development, South West Yorkshire Partnership NHS Foundation Trust, Wakefield WF10 5WE, UK

**Keywords:** preterm infants, metabolomics, metabolic maturation, growth, nutrition, first 1000 days

## Abstract

Growth failure in infants born preterm is a significant issue, increasing the risk of poorer neurodevelopmental outcomes and metabolic syndrome later in life. The aim of this study was to characterize patterns associated with urinary metabolites in extremely preterm and very preterm infants and to explore relationships with growth over time. Untargeted hydrogen-1 nuclear magnetic resonance (^1^H NMR) spectroscopy was used to characterize changes in urinary metabolites over time and explore relationships with growth and nutritional intake. Partial least-squares regression models were constructed to identify metabolic variation associated with age, growth, and nutrition. Biochemical aging differed between very (mean 29.4 weeks ± 1.08 gestational age; *n* = 21) and extremely (≤28 weeks gestational age; *n* = 33) preterm infants, but these differences were not apparent when urinary metabolites were aligned to postmenstrual age. Citrate was the only metabolite significantly positively associated with weight-for-age Z-score. At weeks 1 and 2, citrate was positively associated with gestational age and a greater increase in WAZ from birth to discharge. Biochemical aging aligned to chronological age differed between very and extremely preterm infants, but this variation was lost when aligned to postmenstrual age. Urinary citrate excretion in the first two weeks of life was associated with weight gain and may be a future modifiable biomarker to improve growth outcomes.

## Graphical abstract

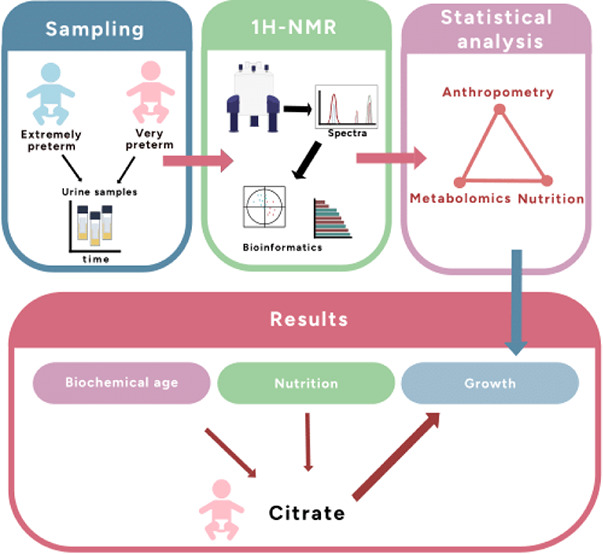

## 1 Introduction

Good growth in infancy is essential for healthy development.^1^ Currently, growth on or around the centile line at birth is recommended for preterm infants, and such growth has been associated with improved short^2,3^ and long-term outcomes.^4^ Despite seemingly adequate nutrition, some infants born preterm continue to experience poor growth.^5^ Reasons for this include prenatal factors such as *in-utero* growth, metabolic immaturity *ex-utero*, inadequate nutritional substrates, genetics and inflammatory conditions.^6^ To overcome these challenges, personalized nutrition support strategies have been proposed to provide the optimum balance of micro- and macronutrients to support growth and achieve better outcomes for this vulnerable population.^6,7^ Biochemical maturation is one such metric against which nutrition could be personalized. This can be defined as the age-dependent development of the metabolic system and can be modeled using metabolomic approaches.^8^ Analytical techniques such as ^1^H nuclear magnetic resonance (NMR) spectroscopy and mass spectrometry (MS), are increasingly used to simultaneously measure a diverse range of metabolites within a biological sample, such as urine, capturing the metabolome in high-resolution.^8,9^ This metabolic profile provides a snapshot of the interacting biochemical processes occurring in the biological system, as well as contributions from environmental sources, such as the diet and the gut microbiota, which collectively exert a strong influence on the developing infant phenotype with implications for health and disease.

A scoping review considering urinary markers of metabolic maturation indicated distinct metabolic profiles associated with prematurity and revealed deficiencies in amino acid, carbohydrate, and fatty acid metabolism pathways in preterm infants at birth. Following birth, levels of glucogenic amino acids, tricarboxylic acid (TCA) cycle metabolites and urinary choline metabolites increase and are correlated with postmenstrual age and gestational age at birth, suggesting a unique preterm pattern of metabolic maturation.^10^ Preterm infants with plateauing or decreasing weight have been shown to have 3-methylhistidine/creatinine ratios above normal range.^11^ Developing phenome-for-age z-scores (PAZ) for these and other metabolites of interest would supplement existing knowledge and be of clinical interest.^8,12^ However, there are gaps in our knowledge relating to (i) the normal pattern of metabolic maturation for preterm infants, (ii) the variation in metabolic signatures among infants with metabolic instability (*e.g.,* intolerance of glucose or lipid) compared to those tolerant of nutrition, (iii) how nutritional interventions could be personalized to support metabolic maturation and improve growth outcomes, and (iv) opportunities to develop reference standards for metabolic maturity, i.e., metabolism may be related to gestational age/postmenstrual age (approximate days since conception), rather than chronological age.^4–6,8,8,10^

In this study we applied an untargeted metabolomic approach to measure metabolites in urine sampled weekly from preterm infants from birth to term equivalent age. These temporal profiles were used to identify age-dependent metabolic variation and understand their relationship with growth and nutritional intake. To explore the impact of prematurity on biochemical aging, extremely (≤28 weeks gestational age) preterm infants were compared to those born very preterm (28 to 32 weeks gestational age).

## 2 Experimental Design

### 2.1 Subject Recruitment

The study was conducted in a tertiary neonatal unit which serves as a regional referral center for surgical, cardiac and other pediatric subspecialties. Infants were eligible for inclusion if they were born at a gestation of less than 32 weeks postmenstrual age (by ultrasound assessment). Infants with surgical problems or who had genetic syndromes expected to impact growth were excluded. Clinical, nutritional and growth data were gathered from multiple clinical information systems and by research nurses as previously described.^4^

### 2.2 Urine Sampling

Urine samples were taken as soon as possible after informed written consent was obtained and then at roughly weekly intervals (Supplementary Figure S1). Urine was collected by placing balls of cotton wool in the diaper. When the cotton wool balls were soaked with urine, they were removed. Soaked cotton wool balls were placed in the barrel of a syringe from which the plunger had been removed. The plunger was then used to squeeze the urine sample out of the cotton wool, through a sterile syringe filter with a pore size of 0.2 mm (Fisherbrand) into a 2 mL centrifuge tube. Samples were stored at −20 °C on the neonatal unit before being transferred to a −80 °C freezer. When a urine sample was contaminated by stool or was less than 500 μL in volume, it was discarded, and a new sample was sought. Data are presented as median and range unless stated otherwise.

### 2.3 ^1^H NMR Spectroscopy

Urine samples (540 mL) were combined with phosphate buffer solution (60 mL) containing the internal standard, 3-trimethylsilyl-1-[2,2,3,3–2H4] propionate (TSP), and sodium azide. Samples were vortexed and spun at 15,000*g* for 10 min before transfer to 5 mm NMR tubes. Spectral profiles were measured on a Bruker 700 MHz NMR spectrometer equipped with a cryoprobe and SampleJet autosampler using a one-dimensional NOESY presaturation pulse sequence. A total of 32 scans were acquired, after 8 dummy scans, and collected in 64K data points. Spectra were automatically phased, baseline corrected and calibrated to TSP (δ 0.0) in Topspin (version 4.3.0). Using in-house scripts, processed spectra were imported into MATLAB (R2023a), redundant spectral regions (e.g., water, urea, TSP peaks) were removed, and manual peak alignment was performed. Urine spectra were normalized using a median fold approach. Peak integration was used to obtain a relative abundance for metabolites of interest.

### 2.4 Data Analysis

Partial least-squares (PLS) regression models were constructed using the ropls R package using either the metabolite matrix or nutrition matrix as the descriptor variable and an individual response variable. PLS discriminant analysis (PLS-DA) models were constructed when a binary variable was used by setting the descriptor variable as a factor. Response variables included chronological age in days, length of pregnancy (in days), change in weight-for-age Z-scores (WAZ) from birth to discharge for the urine metabolites (*x*-axis), and urinary citrate excretion (adjusted for WAZ) as the response variable (*y*-axis) for the nutrition matrix. The predictive ability (Q^2^Y) of each model was calculated using 7-fold cross validation and model validity was confirmed through permutation testing (1,000 permutations, *p* ≤ 0.05). For each model, variable importance (VIP) scores were obtained to identify metabolites or nutrients contributing to the model. Consistent with standards in the field, metabolites with VIP scores greater than one were considered influential to the model.^13^ Where multiple samples were available from an individual at a particular sampling range, a mean of the samples were taken. Splines were produced from the time-series data using the *fda* R package using the function “create.bspline.basis” with an order of 4 to not assume linearity of the data. Separate splines were produced for data from extremely and very preterm infants and permutation testing was used to confirm a significant difference between the extremely and very preterm splines (1000 permutations, *p* < 0.05). Mediation analysis was performed using the package *mediation* in R with 1000 simulations.

## 3 Results

Fifty-four preterm infants were recruited, with a mean GA of 27 weeks +2 days (SD 2.5 weeks, range 23 + 0–31 + 6) and mean birth weight of 900 g (SD 350 g, range 480–2000 g). This study included 33 extremely preterm infants (<28 weeks) with a mean GA of 25^+4^ weeks (SD 1.5 weeks), and 21 very premature infants (≥28 weeks–≤ 32 weeks) with a mean GA of 29^+6^ weeks (SD 1.1 weeks). None of the included infants died before discharge from the study center or had any comorbidities or genetic anomalies that would impact growth. A significant proportion (30%) were transferred to other hospitals before discharge, influencing the range of follow-up periods and number of urine samples for each infant (Table 1).^14^ The approach to nutrition in this cohort has been previously described and focuses on early use of parenteral nutrition and breastmilk fortifier, with donated breastmilk used to supplement maternal breastmilk when required. Supplementary Table 1 describes the median intake of major macronutrients after the first 5 days of life and compares these intakes to the guideline of the European Society of Paediatric Gastroenterology, Hepatology and Nutrition (ESPGHAN). Changes to macronutrient intake during the first 4 weeks of life are illustrated in Supplementary Figure 2.^15^ Macronutrient intakes were similar for very preterm and extremely preterm infants, except for fat intake, which was higher in very preterm infants. After the fifth day of life, macronutrient intakes were compliant with ESPGHAN guidance. Supplementary Figure 3 illustrates the change in weight-for-postmenstrual age z-score in very preterm and extremely preterm infants. For both groups, the z-score declined quickly during the first week of life (very preterm: −0.73, extremely preterm: −0.68) and then much more slowly during the subsequent 5 weeks (very preterm: −0.26, extremely preterm: −0.15). Changes in weight z-scores were not significantly different between the gestation groups.

**Table 1:** Characteristics of Included Infants

Characteristic	Summary Statistic
Gestational age at birth mean ± SD (weeks^+days^)	27 + 2 ± 2.5 (23 + 0–31 + 6)
Birthweight (g) mean ± SD (min,max)	900 ± 350 (480–2000)
Birthweight kg mean ± SD extremely preterm	0.78 kg ± 0.170.78 ± 0.17 kg
Birthweight kg mean ± SD very preterm	0.78 ± 0.17 kg 0.78 ± 0.17 kg
	1.29 kg ± 0.34
Birthweight z-score, mean ± SD	–0.43 ± 0.95
Sex (male), n (%)	22 (41%)
Singleton, n (%)	42 (78%)
Small for gestational age, n (%)	10 (19%)
Complete course of antenatal steroids, n (%)	45 (83%)
Smoking during pregnancy, n (%)	10 (19%)
Index of Multiple Deprivation (IMD) decile^a^, mean ± SD	5.4 ± 2.8
Follow-up period (days)	67 (6–147)
Transferred before discharge home, n (%)	16 (30%)
Number of samples collected, median ± SD	6 ± 3.29
Number of urine samples collected (extremely preterm), median ± SD	7 ± 3.35
Number of urine samples collected (very preterm), median ± SD	4 ± 2.62

a IMD (1 = most deprived, 10 = most affluent with higher deciles being less deprived).

### 3.1 Biochemical Aging Differs between Very and Extremely Preterm Infants

Mean weight at birth in extremely preterm infants was 0.78 ± 0.17 kg compared to 1.29 ± 0.34 kg in very preterm infants (*p* < 0.05). (Figure 1a). However, there was no statistical difference in the change in WAZ score from birth to discharge for extremely preterm infants compared to very preterm infants (Figure 1a).

**Figure 1:**
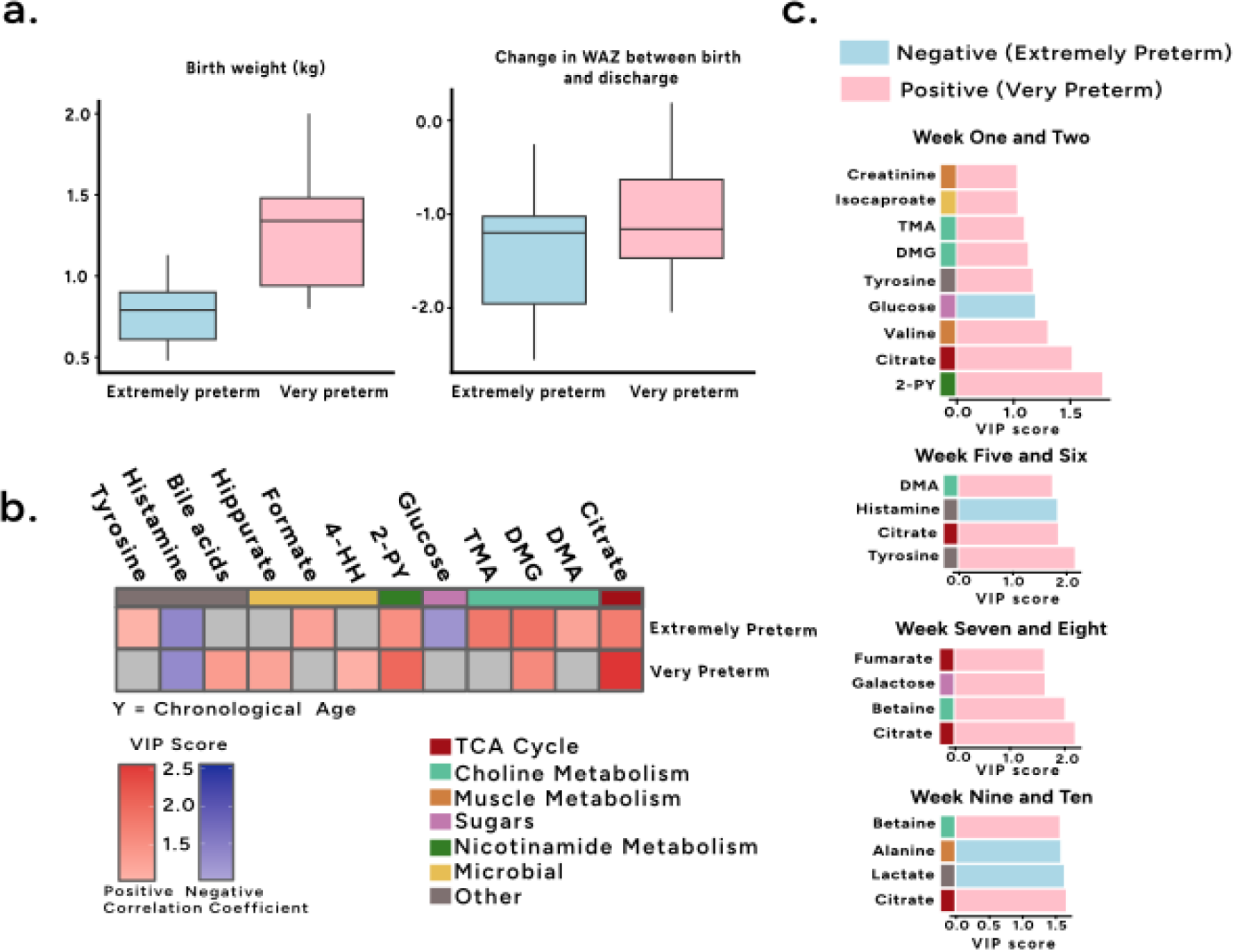
Differences in biochemical aging between very and extremely preterm infants. (a) Box plots comparing birth weight (kg) and change in weight-for-age Z (WAZ) scores from birth to discharge in very preterm and extremely preterm infants. (b) Heatmap highlighting the significant discriminatory metabolites (VIP > 1; correlation with age *p* < 0.05) from the PLS models identifying biochemical variation associated with age. Separate models were constructed for the very and extremely preterm infants. (c) PLSDA models comparing the urinary metabolic profiles of very and extremely preterm infants at biweekly sampling points. Metabolites shown are those that passed the criteria for significance (VIP > 1; correlation with age *p* < 0.05).

Age-dependent biochemical variation was explored in the urinary profiles from extremely and very preterm infants independently. Here, a PLS model was constructed using the urinary metabolites as the descriptor matrix and chronological age (in days) as the response variable. A significant model was obtained for extremely preterm infants (Q^2^Y = 0.24; *p* = 0.001). Considering only metabolites with a variable importance (VIP) score greater than 1 (and a correlation significance with chronological age <0.05), citrate, dimethylglycine (DMG), *N*-methyl-2-pyridone-5-carboxamide (2-PY), dimethylamine (DMA), trimethylamine (TMA), formate, and tyrosine increased with age, while the excretion of glucose and histamine decreased (Figure 1b). Similarly, a significant PLS model was obtained for very preterm infants (Q^2^Y = 0.33; *p* = 0.001). Consistent with extremely preterm infants, citrate, DMG and 2-PY increased with age, in addition to hippurate, 4-hydroxyhippurate (4-HH), and spectral resonances arising from bile acids, while histamine decreased with age (Figure 1b).

The urinary metabolic profiles of extremely and very preterm infants were compared at each sampling point via PLS-DA models (Figure 1c). At 1–2 weeks, there were statistically significantly higher amounts of glucose noted in the extremely preterm (*n* = 12) compared with higher amounts of citrate, DMG, 2-PY, TMA, creatinine, tyrosine and isocaproate observed in the very preterm infants (*n* = 33) (Q^2^Y = 0.24; *p* = 0.001). No statistically significant differences were observed at weeks 3–4. At weeks 5–6, the extremely preterm infants (*n* = 10) excreted greater amounts of histamine and the very preterm infants (*n* = 30) excreted greater tyrosine, DMA and citrate (Q^2^Y = 0.11; *p* = 0.041). At 7–8 weeks, there were no metabolites significantly associated with extremely preterm infants (*n* = 23), but excretion of citrate, betaine, galactose and fumarate was significantly higher in very preterm infants (*n* = 9) (Q^2^Y = 0.11; *p* = 0.046). At weeks 9–10, higher urinary alanine and lactate was noted in those born extremely preterm (*n* = 19) compared to higher amounts of betaine and citrate observed in the very preterm infants (*n* = 6) (Q^2^Y = 0.21; *p* = 0.047) (Supplementary Table S2).

### 3.2 Biochemical Age of Urinary Profiles for Extremely and Very Preterm Infants Do Not Differ When Aligned to Postmenstrual Age

To explore the relationship between prematurity and biochemical aging, variation was explored in metabolites identified to exhibit age-dependent patterns. These metabolites were compared between extremely and very preterm infants based on day of life (days since birth) and postmenstrual age (days since mother’s last menstrual period as assessed by ultrasound scanning at the end of the first trimester of pregnancy). This was analyzed through the production of splines for each individual followed by permutation testing to determine significance between the extremely preterm and very preterm groups (Figure 2; Supplementary Figure 3). Based on chronological age, a significant difference was observed between citrate, tyrosine and DMG excretion over the first 14 weeks of life between the two groups, being higher in very preterm infants compared to extremely preterm infants. A significant difference in glucose excretion between extremely and very preterm infants was also observed, being significantly higher in extremely preterm infants (*p* = 0.001). However, this difference was lost when the urinary profiles were aligned by postmenstrual age. The higher excretion of glucose was not related to glucose/carbohydrate intake in these infants (Supplementary Figure S4, Supplementary Figure S5 and Supplementary Table S3).

**Figure 2:**
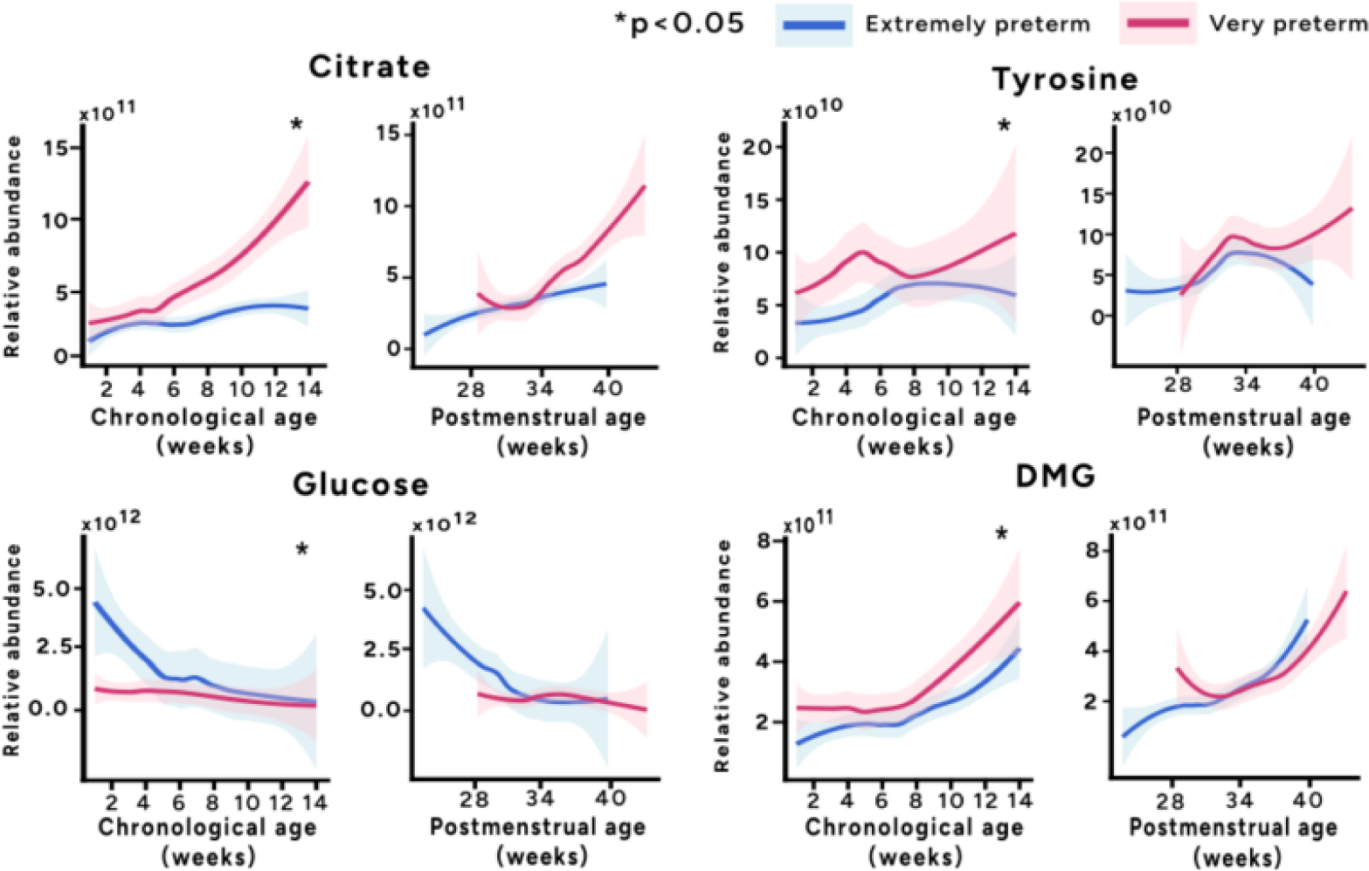
Comparing biochemical aging based on chronological or postmenstrual age. Variation in the relative abundance of urinary citrate, tyrosine, glucose and dimethylglycine (DMG) across extremely and very preterm infants with age. Splines were produced for individuals and compared between the groups to determine significance (*p* < 0.05, 1000 permutations).

### 3.3 Early Urinary Citrate Excretion is Positively Associated with Weight Gain and is Modulated by Nutritional Factors

To identify urinary metabolites that may predict weight changes in these infants, PLS models were constructed. Here, the urinary metabolites measured at all time points served as the descriptor matrix, adjusted for sampling day of life and gestational age covariates, and change in WAZ between birth and discharge served as the response variable (Q^2^Y = 0.0042, *p* = 0.045). From this model, citrate was the only metabolite with a VIP score greater than 1 and a *p* < 0.05, being positively correlated with WAZ. Figure 3a highlights that citrate excretion at week 1 and 2 is positively associated with gestational age. Figure 3b indicates higher citrate excretion is associated with a greater increase in WAZ from birth to discharge. Mediation analysis was performed to explore the causal mediation effect of BGA on change in WAZ between birth and discharge through citrate. This analysis identified citrate as a significant but modest mediator in this effect (*p* = 0.022, 10.3% proportion mediated, Supplementary Table S5).

**Figure 3:**
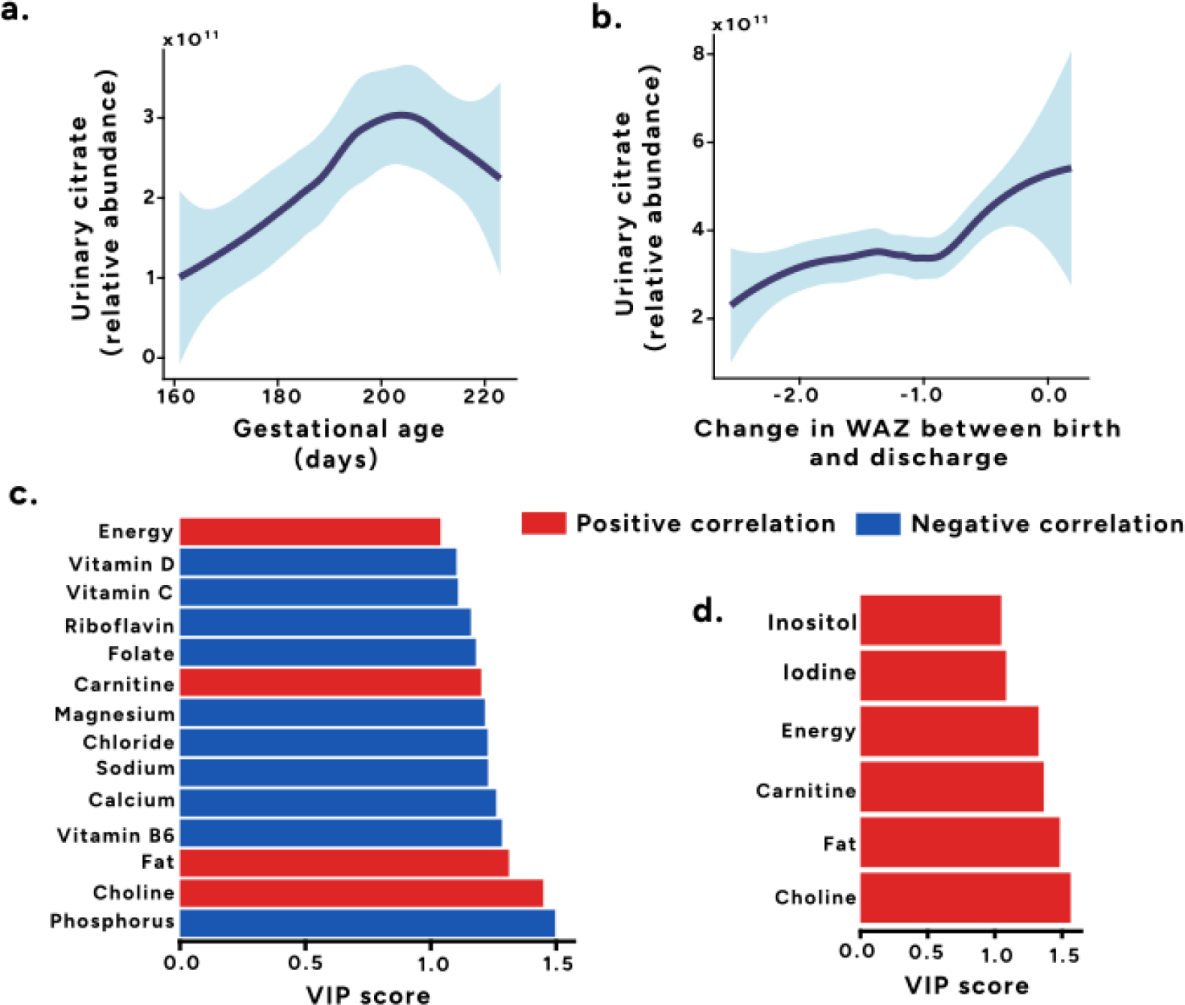
Early citrate excretion is positively associated with weight gain and is modulated by nutritional factors. Relationship between citrate excretion in first 2 weeks of life and (a) gestational age at birth, and (b) change in weight-for-age Z-score (WAZ) between birth and discharge. (c) Variable Importance in Projection (VIP) plot from the PLS model identifying nutritional components associated with citrate excretion adjusted by chronological age and gestational age (VIP > 1, *p* < 0.05). (d) VIP plot from the PLS model identifying nutritional components adjusted by WAZ, chronological age and gestational age associated with urinary citrate excretion (VIP > 1, *p* < 0.05).

As urinary citrate was positively related to weight changes, nutritional factors influencing citrate excretion were investigated (Figure 3c and d). Nutritional profiles comprising 33 nutritional measures (Supplementary Table S4) were calculated for each infant. A PLS model was built using these nutritional profiles as the descriptor matrix and the relative abundance of urinary citrate as the response variable. As nutritional intake varied based on prematurity and age, the nutritional profiles were adjusted for chronological age and gestational age. Based on VIP > 1 and *p* < 0.05, dietary choline, fat and energy intake were positively associated with urinary citrate excretion and phosphorus, vitamin B6, calcium, sodium, chloride, magnesium, folate, riboflavin, vitamin C and vitamin D were negatively associated with urinary citrate excretion. Another PLS model was built with nutritional profiles adjusted by WAZ, chronological age and gestational age using urinary citrate excretion as the response variable. Here, choline, fat, carnitine, energy, iodine and inositol intake were all positively associated with urinary citrate excretion (VIP > 1, *p* < 0.05).

## 4 Discussion

In this study, we show that biochemical aging differed between very and extremely preterm infants when aligned by chronological age, however, these differences are lost when urinary metabolites are adjusted to postmenstrual age. This indicates that postmenstrual age is a more important consideration in terms of biochemical demands against which nutritional strategies can be targeted rather than chronological age (e.g., time since birth). Our findings demonstrate that biochemical variation exists across infants based upon their degree of immaturity, emphasizing the need for more specific nutritional care pathways based on gestational age and maturity, rather than blanket recommendations based on birthweight alone.

Based on chronological age, a significant difference was observed between citrate, tyrosine and DMG excretion over the first 14 weeks of life between the two groups, being higher in very preterm infants compared to extremely preterm infants. Specifically, early citrate excretion, notably in the first 2 weeks of life, was positively associated with weight gain over time. This indicates that urinary citrate, an intermediate in the energy-generating TCA cycle, is a potential early putative marker for later growth. This is consistent with the high energy demands required for growth across the first year of life, ranging from 40% in the first month to 17.5% in the next three months. As such, urinary citrate may reflect the energy generating capacity of an infant and therefore their propensity to grow. Adjusting nutrition profiles for age and prematurity identified that dietary choline, fat and energy intake were positively associated with citrate excretion, while adjusting by WAZ and age found that choline, fat, carnitine, energy, iodine and inositol were positively related with urinary citrate. Given that growth requires both nutrients and energy to allow tissue accretion, associations with increases in TCA cycle metabolites secondary to increased energy utilization would seem plausible. In contrast, urinary citrate was negatively associated with the intake of nutrients such as phosphorus. This highlights nutritional factors can potentially modify energy generating pathways in the infant and ultimately growth.

Although the decision to initiate or progress enteral feeds is subject to multiple factors (e.g., clinical status, concurrent infection or abdominal concerns along with clinician preferences), most infants will have transitioned from parenteral nutrition to be fully enterally fed around 2-weeks of age. In this work we did not observe significant changes in the metabolome following the transition to enteral feeds. This suggests postmenstrual age, rather than chronological age, is a better predictor of changes in metabolome, suggesting timing of transition to enteral feeds is not a contributing factor. In support of these findings, previous studies show that postmenstrual age in preterm infants is associated with variations in urinary metabolomic profiles over time^16–18^ reflecting metabolic maturation and organ development.^19^ Rapid postnatal adaptation of the TCA cycle, along with energy and carbohydrate metabolism, occurs with the emergence of gluconeogenic enzymes to support neonatal glucose homeostasis.^19,20^ Other studies have also shown postnatal changes to the urinary metabolome of preterm and term infants over time, with increases in urinary metabolites associated with the TCA cycle, including citrate. These changes were associated with increases in length and weight gain^21^ and higher energy requirements during periods of rapid growth.^19,22^ Moltu et al. explored the effect of different nutritional interventions on metabolic maturation in preterm infants. Preterm infants with a birth weight of <1500 g were randomized to receive enhanced nutrition intake (average intake of 139 kcal/kg/day and 4 g/kg/day protein) compared to a standard diet (average intake of 126 kcal/kg/day and 3.2 g/kg/day protein). Results showed significant changes over time in the urinary metabolome during the early postnatal period in preterm infants, especially for glucogenic amino acids and TCA cycle metabolites, suggesting high metabolic turnover relative to energy metabolism and amino acid synthesis, supporting our present findings. Interestingly, Moltu et al.^19^ did not report any differences in urinary metabolome between infants who received enhanced nutrition and displayed better growth, and those who did not. There was also no association between nutrient intakes and the urinary metabolome. However, these findings may have been impacted by the increased incidence of hypophosphatemia (77% vs 26%) and hypokalemia (88% vs 46%) in the enhanced nutrient intake group in the first week of life. These deficiencies would be expected to have a downstream effect on the TCA cycle^23^ and negative associations with weight and age.

Hao et al.^24^ suggested that some of the changes seen in the preterm infant urinary metabolome may be due to defects in the metabolism of macronutrients when compared to term infants. The abundance of the amino acids lysine, phenylalanine, histidine, ornithine, fumarate, and malate in the urine of preterm infants were significantly lower than that of full-term infants, with some at undetectable levels. Lower excretion of amino acids in preterm infants may reflect insufficient intake or increased utilization of these molecules for catch up growth. Similarly, the same study found reduced urinary lactose, stearic acid and succinate. The reduction in lactose (a carbohydrate) and stearic acid (a fatty acid) suggests either insufficient intake of these nutrients, or increased utilization associated with growth meaning that they do not appear in the urine. Succinate, like citrate, is generated in the TCA cycle and is marker of energy metabolism; it is unclear if this change is due to reduced precursors or enzymes, or reduced energy metabolism or as a consequence of inadequate intake of certain nutrients.^12^ Taken together, these studies suggest a complex relationship between nutrient intake, growth and urinary metabolome, extending beyond the effects of prematurity itself. An example of this is described in the elegant study by Priante et al.^25^ where the distinctive metabolic urinary profiles of very preterm (<32 weeks) infants with intrauterine growth restriction (IUGR) were described within the 48 h following birth and compared with nongrowth restricted preterm infants. IUGR preterm infants had derangements in pathways associated with glucocorticoids, sex hormone biosynthesis, tryptophan-kynurenine, and methionine-cysteine pathways compared to non-growth restricted preterm infants, which may explain aberrant intrauterine growth. Infants with IUGR often have poorer postnatal growth and long-term outcomes, and using tailored nutritional support may improve longer term outcomes in this vulnerable cohort.^25^

There is evidence to suggest this proposed process of metabolic maturation continues throughout infancy and is not restricted to the preterm period. Importantly, changes in metabolic maturity and development may be associated with outcomes. In malnourished children, immature metabolic development is associated with growth faltering.^8^ Giallourou et al.^8^ used urinary metabolic profiling to study metabolic maturation of infants from resource constrained settings over three continents in the first 1000 days of life. Age-specific reference curves for the eight most commonly perturbed urinary metabolites (independent of feeding practices) in malnourished children were obtained and from which phenome-for-age z-scores (PAZ) for each child (a measure of biochemical maturity) were developed.^7^ This work suggests that evolving metabolic demands during infancy and early childhood, especially time-dependent changes in energy- and choline-related metabolites, molecules arising from nutrient-gut microbial-host metabolic interactions, and muscle-associated compounds.^7,8^

The use of urinary metabolomics may offer an objective window into the maturing biochemical demands of each infant; providing the opportunity to design personalized nutrition support based on metabolic maturation and function, improving growth and other outcomes. Identifying and exploring metabolites of interest that may be associated with growth and other outcomes in this vulnerable cohort would be of value. Interestingly patterns of urinary citrate in preterm infants in this study (Figure 2) are similar to those described by Nilsson et al.^26^ The results of these studies suggest metabolic maturity is related to postmenstrual age and not chronological age, and this has potentially important implications for the nutritional care of preterm infants. In support of this concept, evidence suggests that more immature preterm infants are more likely to encounter metabolic problems and do not respond or grow as expected in the face of reasonable amounts of nutrition.^10^ Chacko and Sunehag^27^ have previously demonstrated that preterm infants continue to generate glucose via gluconeogenesis in the face of sufficient glucose supply. This process is independent of blood glucose and insulin levels, supporting the idea that the phenomenon is driven by prematurity itself. Further to this, there may be metabolic differences between preterm infants born small or large for gestational age, and so urinary metabolomics may help to personalize nutrition support for unusually small or large infants.^28^ Many very preterm and extremely preterm infants (born before 32 weeks gestation) will be managed in similar ways from a nutritional point of view. Most international guidelines and recommendations for nutrient intakes and nutritional management refer to all infants below a given birthweight or gestation without discriminating between smaller or most premature infants. Current ESPGHAN parenteral and enteral recommendations for preterm infants essentially cover all infants with a birthweight below 1800 g.^15,29–31^ Similarly, current AAP guidance considers all preterm infants below 1500 g to be similar, though it does make additional recommendations for those born <1000 g.^32^ None of these guidelines goes further to give specific recommendations or considerations for the most extremely preterm infants or those below 800 g.

### 4.1 Limitations and Strengths

There are several limitations with this study including the relatively small sample size, lack of paired samples over time, and a lack of a term reference group restricting our interpretation of biochemical maturity to a preterm context. Furthermore, missing length data points made the interpretation of growth patterns difficult. As a result, the potential for selection bias cannot be excluded, and as our cohort was drawn from a single clinical site, it limits the generalizability of the metabolic signatures identified. Similarly, not every included infant had a urine sample at every time point, resulting in a degree of missing data for the longitudinal metabolomic analysis. In addition, in this analysis NMR spectral peaks arising from bile acids were integrated providing a measure of relative abundance of this broad metabolite class. However, with this approach it is not possible to distinguish individual bile acid species. As such, the general label of bile acids was used and is a limitation of this work. A larger sample size will be required to reliably identify metabolites associated with metabolic stability and growth, with a view to developing phenome age z-scores or identify biomarkers of growth. However, a strength of this study is detailed longitudinal data and nutrient intake and growth to accompany the rich urinary metabolic data.

## 5 Conclusions

Our results show that biochemical aging of the urinary metabolome changed in response to postmenstrual age rather than gestation, making a case for recommendations for nutritional care in these vulnerable infants to be based more closely on gestational age/postmenstrual age, and perhaps more specific brackets of prematurity, rather than birth weight. Nutrient profiles adjusted for age and prematurity showed that urinary citrate excretion was positively associated with choline, fat and energy intake. This contrasted with negative associations between urinary citrate excretion and nutrients such as phosphorus. Early citrate excretion in the urine was positively associated with weight gain, suggesting that urinary citrate may be an important early biomarker of nutritional adequacy and metabolic maturation and predictor for later growth. Future work should consider the development of standardized urinary reference ranges for metabolites of interest, such as citrate, to explore whether urinary citrate monitoring could be used to improve growth and other important outcomes, as part of personalized nutrition support.

## Supplementary Material



## Data Availability

Data are available on MetaboLights: MTBLS12794 (ID: REQ20250722211998) https://www.ebi.ac.uk/metabolights/reviewerfd2e1c75-0f7d-4636-a5a3-e3cd82017162

## Notes

Supplementary Table S1: mean intake of macronutrients between day 6 and day 42 of life compared to ESPGHAN-recommended intake for preterm infants (2022); Supplementary Table S2: PLS models summary; Supplementary Table S3: *P*-values for fda calculations; Supplementary Table S4: nutritional measures; Supplementary Table S5: mediation analysis results; Supplementary Figure S1: plot of each individual infant’s samples at different postmenstrual ages; Supplementary Figure S2: macronutrient intakes in extremely preterm and very preterm infants during the first 4 weeks of life; Supplementary Figure S3: change in weight-for-postmenstrual age z-score separated by gestation group; Supplementary Figure S4: heatmap showing significant results of the PLS model (VIP > 1, *p* < 0.05) of biochemical aging, where the metabolite matrix has been adjusted by sex; Supplementary Figure S5: graphs of infants at weeks 1 and 2 showing changes in urinary excretion of tyrosine, glucose, and DMG as gestational age increases; Supplementary Figure S6: graph showing urinary glucose excretion against the amount of carbohydrate given; Supplementary Figure S7: ^1^H NMR urine spectrum annotated with integrated peaks (PDF)

## Notes

The study was conducted in accordance with the Declaration of Helsinki and approved by the NRES Committee South Central-Oxford A (14/SC/1275) (08/09/2014). Informed consent was obtained from all subjects involved in the study. Infants were recruited from the Neonatal Unit of University Hospital Southampton NHS Foundation Trust as part of the Growth Assessment of Preterm Infants Study (GAP) after written informed parental consent.

## Abbreviations

2-PY: *N*-methyl-2-pyridone-5-carboxamide
4-HH: 4-hydroxyhippurate
AAP: American Academy Pedaitrics
DMA: dimethylamine
DMG: dimethylglycine
ESPGHAN: European Society of Paediatric Gastroenterology, Hepatology and Nutrition
^1^H NMR spectroscopy: hydrogen-1 nuclear magnetic resonance spectroscopy
IUGR: intrauterine growth restriction
G: grammes
GA: gestational age
kg: kilograms
kcal: calories
Mmol: millimoles
P: probability
PLS: partial least-squares
SD: standard deviation
TMA: trimethylamine
TCA: tricarboxylic acid cycle
VIP: variable importance
μg: micrcograms
WAZ: weight-for-age Z-score
