# Supplementary material for: Impact of Prematurity on Metabolic Maturation
Source: J Proteome Res. 2026 Mar 23;25(4):1855–63. doi: 10.1021/acs.jproteome.5c00640 (PMC13054858; doi:10.1021/acs.jproteome.5c00640)
Supplement: Supplementary file 1 [file pr5c00640_si_001.pdf]

Supplementary files

Impact of prematurity on metabolic maturation

Kate Pearse [1#], Aneurin Young [1,2#], Mark J Johnson [1,2,4*], R Mark Beattie [3,4,6], Jonathan R. Swann[1,4,6~] and Luise V. Marino [5,7~]

[1] School of Human Development and Health, Faculty of Medicine, University of Southampton, SO16 6YD
[2] Department of Neonatal Medicine, Princess Anne Hospital, University Hospital Southampton NHS Foundation Trust, Southampton UK, SO16 5YA m.johnson@soton.ac.uk
[3] Paediatric Gastroenterology, Southampton Children's Hospital, University Hospital Southampton NHS Foundation Trust, Southampton, UK, SO16 6YD
[4] Southampton NIHR Biomedical Research Centre, University Hospital Southampton NHS Foundation Trust, Southampton UK, SO16 6YD
[5] Faculty of Health Science, University of Southampton, Southampton, UK, SO17 1BJ
[6] Department of Metabolism, Digestion and Reproduction, Imperial College London, UK, SW7 2AZ
[7] Research & Development, South West Yorkshire Partnership NHS Foundation Trust, Wakefield, UK, WF10 5WE

# joint first author, ~joint senior author, *corresponding author

Table of Contents

- **Supplementary Table S1:** Mean intake of macronutrients between day 6 and day 42 of life compared to ESPGHAN recommended intake for preterm infants (2022)
- **Supplementary Table S2:** PLS models summary
- **Supplementary Table S3:** P values for fda calculations
- **Supplementary Table S4:** Nutritional measures
- **Supplementary Table S5:** Mediation analysis results
- **Supplementary Figure S1:** Plot of each individual infant's samples at different postmenstrual ages.
- **Supplementary Figure S2:** Macronutrient intakes in extremely preterm and very preterm infants during the first four weeks of life.
- **Supplementary Figure S3:** Change in weight-for-postmenstrual age z-score separated by gestation group.
- **Supplementary Figure S4:** Heatmap showing results significant results of PLS model (VIP>1, p<0.05) of biochemical ageing where the metabolite matrix has been adjusted by sex.
- **Supplementary Figure S5:** Graphs of infants at weeks 1 and 2 showing changes in urinary excretion of tyrosine, glucose and DMG as gestational age increases.
- **Supplementary Figure S6:** Graph showing urinary glucose excretion against the amount of carbohydrate given.

- **Supplementary Figure S7:** 1H-NMR urine spectrum annotated with integrated peaks

Supplementary Table S1: Mean intake of macronutrients between day 6 and day 42 of life compared to ESPGHAN recommended intake for preterm infants (2022). ** Statistically significant difference between very preterm and extremely preterm infants (p<0.01 using a linear regression model for the effect of gestation group on intake, using subject identity as a random effect)

Nutrient	Median daily intake <28 weeks (IQR)	Median daily intake 28 - >32 weeks (IQR)	Median daily intake – all infants (IQR)	Recommended intake range*
Energy (kcal/kg/day)	117 (101 – 130)	122 (113 – 128)	120 (105 - 129)	115 – 140
Protein (g/kg/day)	3.4 (2.8 – 3.8)	3.6 (3.1 – 3.8)	3.5 (3.0 - 3.8)	3.5 - 4.0
Carbohydrate (g/kg/day)	13.9 (12.1 – 15.4)	14.3 (12.7 – 15.)	14.1 (12.3 – 15.3)	11 – 15
Fat (g/kg/day)	**5.3 (3.8 – 6.2)****	**6.0 (5.6 – 6.3)****	5.7 (4.5 – 6.2)	4.8 - 8.1
Protein Energy Ratio (g/100 kcal)	2.9 (2.7 – 3.1)	2.9 (2.8 – 2.9)	2.9 (2.7 – 3.0)	2.8 - 3.6

Supplementary Table S2: PLS models summary

Model	Number of samples	R^2X	R^2Y	Q^2Y	pQ^2
**All time points**					
Very preterm	94	0.149	0.419	0.333	0.001
Extremely preterm	229	0.330	0.243	0.243	0.001
**Week splits**					
One and two	45	0.236	0.410	0.272	0.001
Three and four	43	0.718	0.347	0.134	0.054
Five and six	40	0.129	0.393	0.112	0.041
Seven and eight	32	0.135	0.448	0.112	0.046
Nine and ten	25	0.133	0.589	0.209	0.047
**WAZ**	323	0.479	0.101	0.0364	0.001
**Citrate**	321	0.553	0.100	0.00419	0.045
**Nutritional model**					
WAZ score	321	0.456	0.0316	0.0216	0.003
Change in WAZ score	318	0.446	0.177	0.170	0.001

Supplementary Table S3: P values for fda calculations

	p values	
Metabolite	Chronological age	Postmenstrual age
Citrate	0.001	1.000
Glucose	0.001	1.000
Tyrosine	0.001	1.000
DMG	0.001	1.000

Supplementary Table S4: Nutritional measures

Nutritional measure	Units
Energy	kcal/kg
Protein	g/kg
Carbohydrate	g/kg
Fat	g/kg
Sodium	mmol/kg
Chloride	mmol/kg
Potassium	mmol/kg
Calcium	mmol/kg
Phosphorous	mmol/kg
Magnesium	mmol/kg
Iron	µmol/kg
Zinc	µmol/kg
Copper	µmol/kg
Selenium	nmol/kg
Iodine	nmol/kg
Manganese	nmol/kg
Vitamin A	International units/kg
Vitamin D	International units/kg
Vitamin E	International units/kg
Vitamin K	International units/kg
Thiamin	µg/kg
Riboflavin	µg/kg
Vitamin B6	µg/kg
Folate	µg/kg

Vitamin B12	µg/kg
Biotin	µg/kg
Pantothenic acid	mg/kg
Niacin	mg/kg
Vitamin C	mg/kg
Taurine	mg/kg
Choline	mg/kg
Carnitine	mg/kg
Inositol	mg/kg

Supplementary Table S5 – Mediation analysis results

Mediation analysis performed using the R-package '*mediation*'. X = BGA, Y = change in WAZ, M = citrate excretion

	ACME				ADE				Total effect				Proportion mediated			
Group	Estimate	95% CI lower	95% CI upper	p-value	Estimate	95% CI lower	95% CI upper	p-value	Estimate	95% CI lower	95% CI upper	p-value	Estimate	95% CI lower	95% CI upper	p-value
All	0.00112	0.000209	0.00213	0.022	0.0097	0.00652	0.0128	2.00E-16	0.0108	0.00762	0.0139	2.00E-16	0.103	0.0194	0.203	0.022

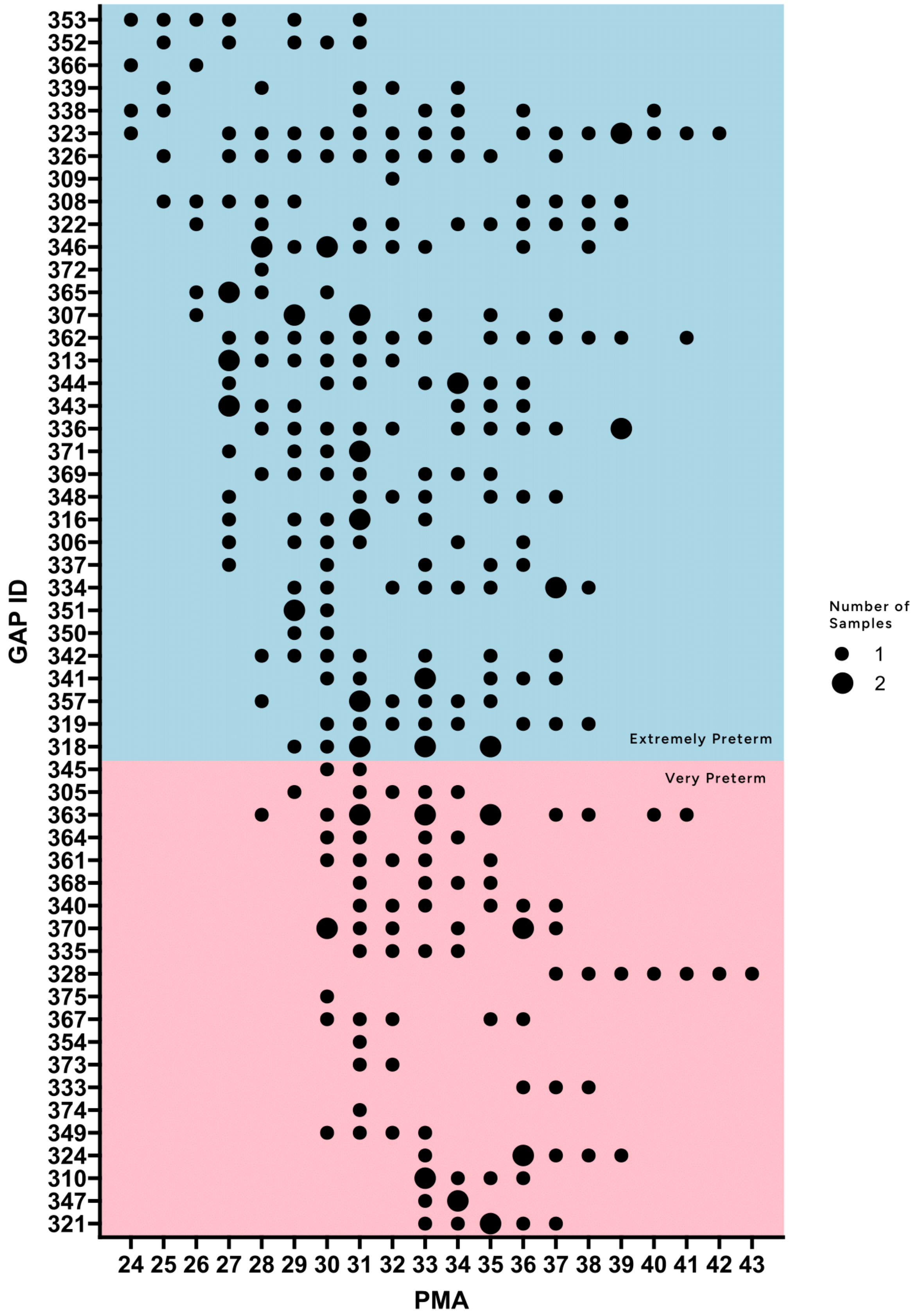

Supplementary Figure S1. Plot of each individual infant's samples at different postmenstrual ages.

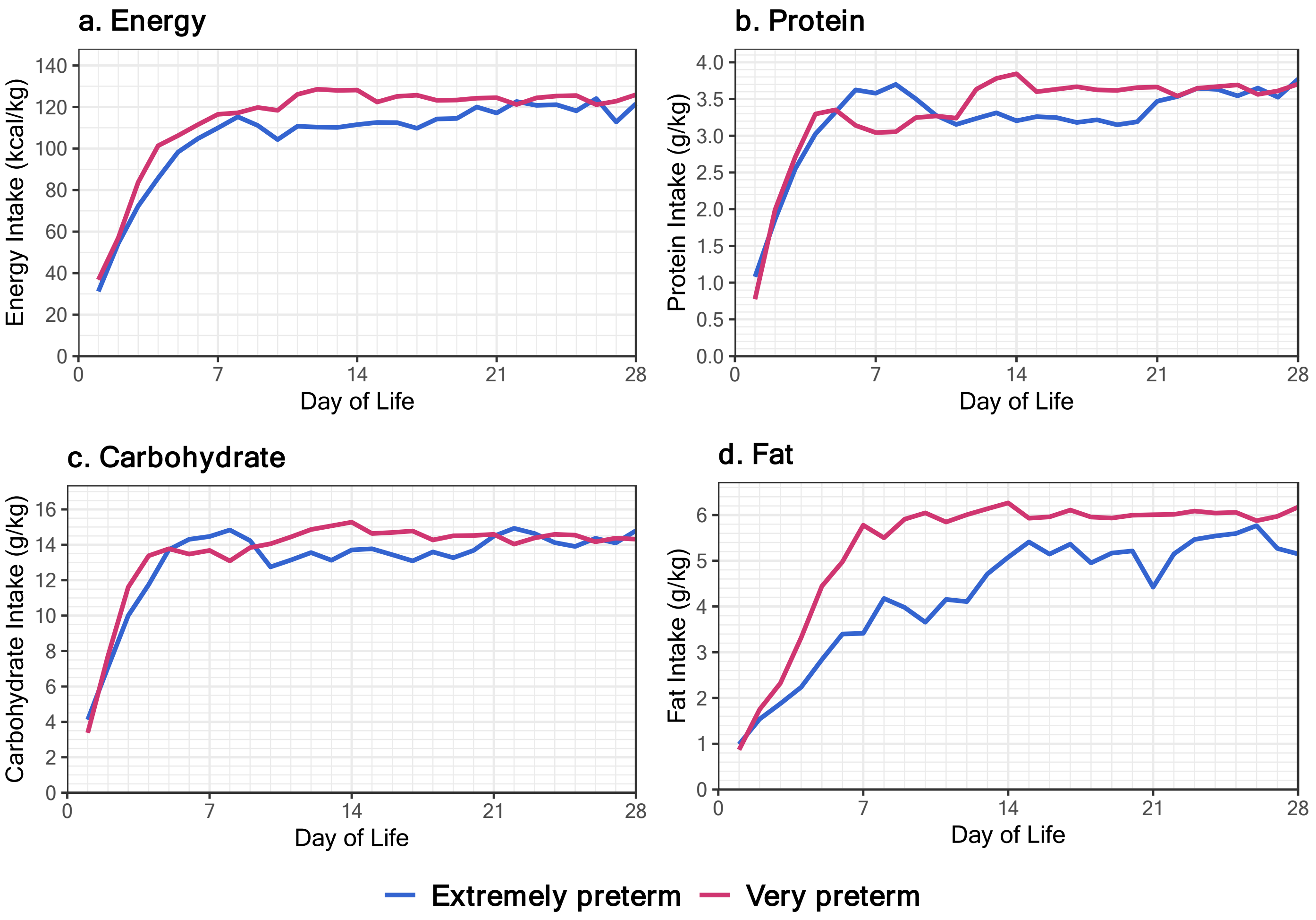

Supplementary Figure S2. Macronutrient intakes in extremely preterm and very preterm infants during the first four weeks of life. Line indicates the median intake for (a) Energy, (b)Protein, (c) Carbohydrate, (d) Fat.

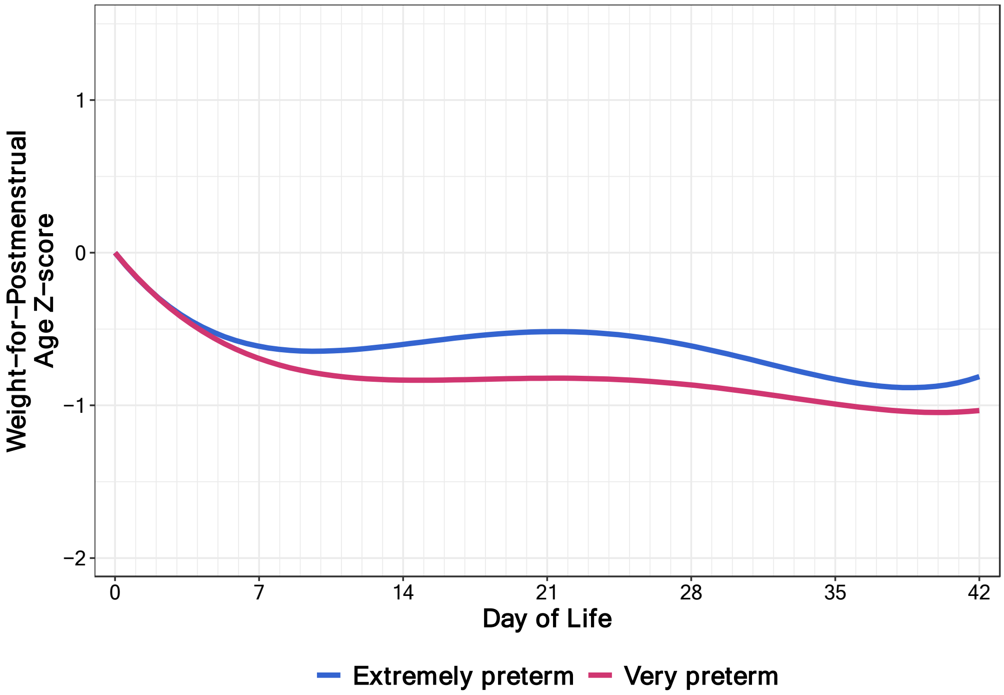

Supplementary Figure S3: Change in weight-for-postmenstrual age z-score separated by gestation group. Thin lines represent the change in z-score for individual infants and thick lines illustrate the trend in z-score change for each gestation group.

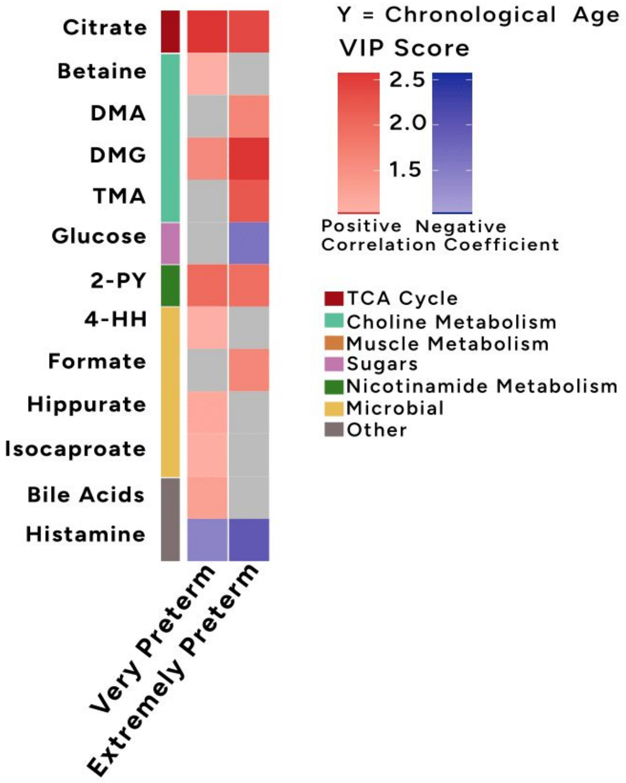

Supplementary Figure S4: Heatmap showing results significant results of PLS model (VIP>1, $p<0.05$) of biochemical ageing where the metabolite matrix has been adjusted by sex.

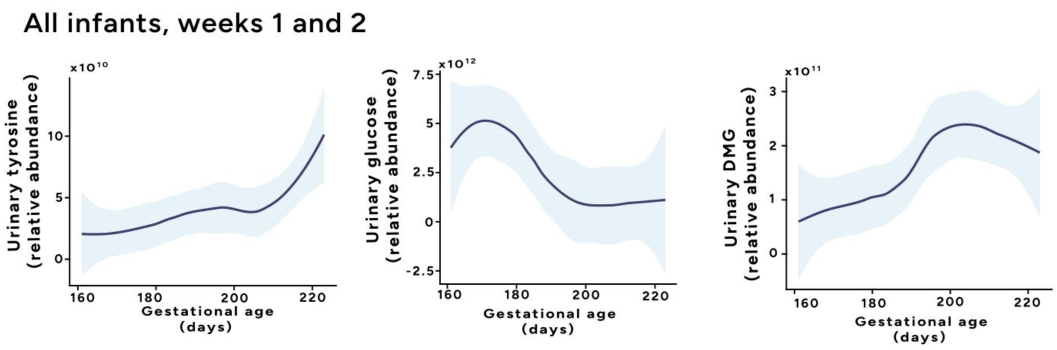

Supplementary Figure S5. Graphs of infants at weeks 1 and 2 showing changes in urinary excretion of tyrosine, glucose and DMG as gestational age increases.

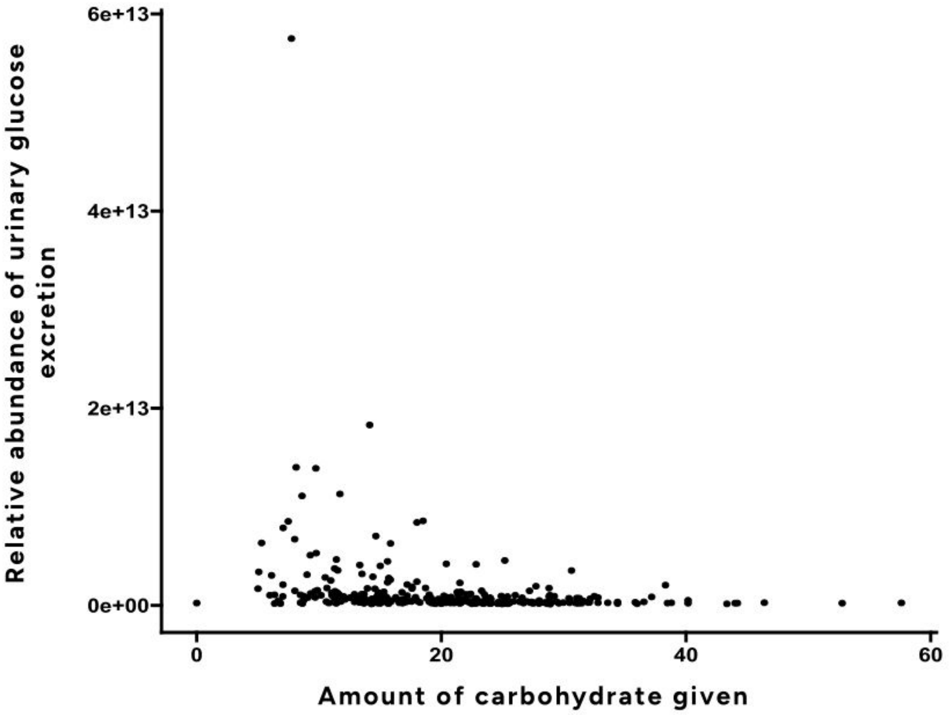

Supplementary Figure S6. Graph showing urinary glucose excretion against the amount of carbohydrate given.

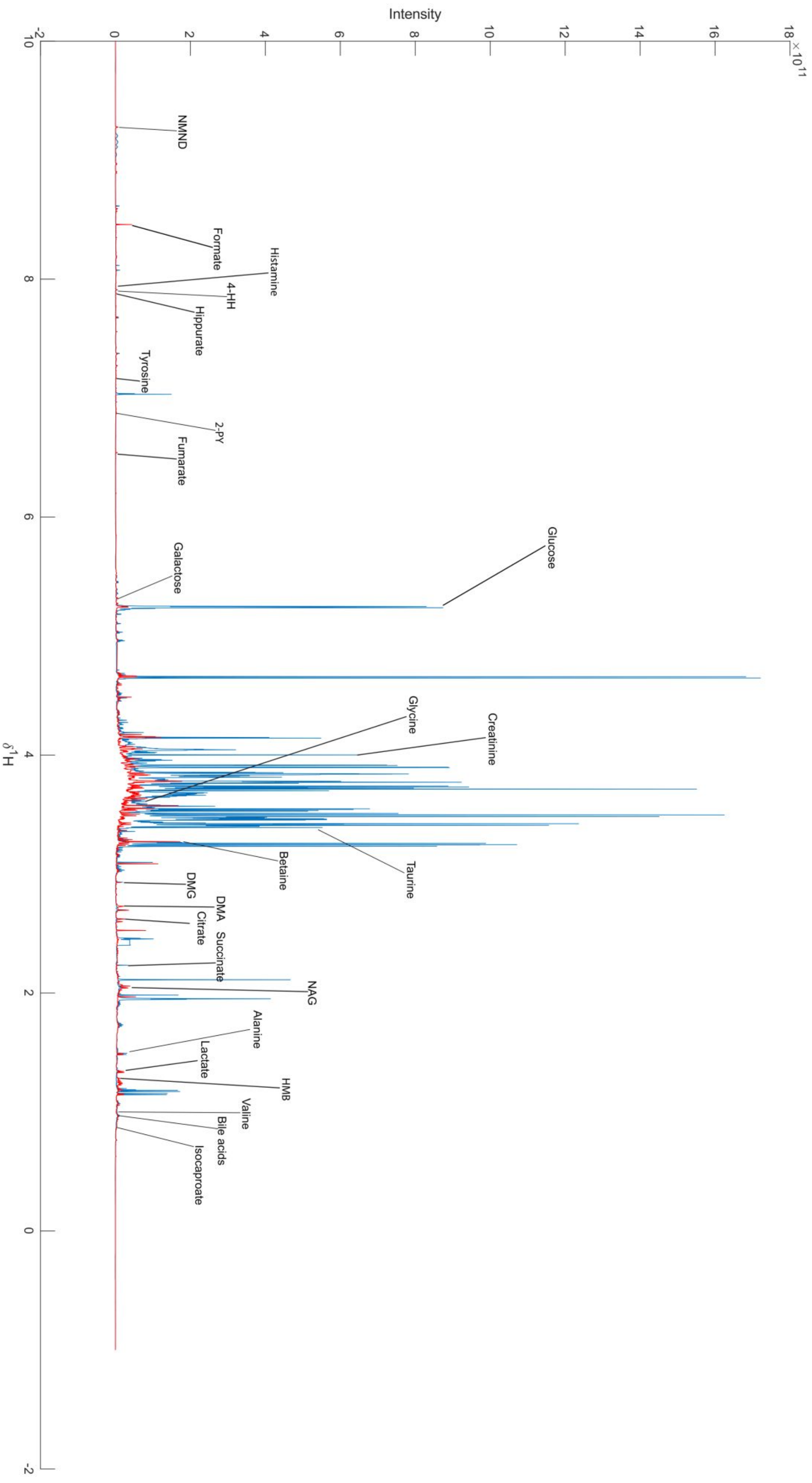

Supplementary Figure S7. 1H-NMR urine spectrum annotated with integrated peaks
